# Survival Ethics in the Real World: The Research University and Sustainable Development

**DOI:** 10.1007/s11948-013-9441-8

**Published:** 2013-04-03

**Authors:** Charles Verharen, John Tharakan, Flordeliz Bugarin, Joseph Fortunak, Gada Kadoda, George Middendorf

**Affiliations:** 1 Department of Philosophy, Howard University, Washington, DC 20059 USA; 2 Department of Chemical Engineering, Howard University, Washington, DC 20059 USA; 3 Department of Sociology and Anthropology, Howard University, Washington, DC 20059 USA; 4 Departments of Chemistry and Pharmaceutical Sciences, Howard University, Washington, DC 20059 USA; 5 Department of Computer Sciences, University of Khartoum, Khartoum, Sudan; 6 Department of Biology, Howard University, Washington, DC 20059 USA

**Keywords:** Interdisciplinary research, Sustainable development, Survival ethics model, Service learning, Howard University, African universities

## Abstract

We discuss how academically-based interdisciplinary teams can address the extreme challenges of the world’s poorest by increasing access to the basic necessities of life. The essay’s first part illustrates the evolving commitment of research universities to develop ethical solutions for populations whose survival is at risk and whose quality of life is deeply impaired. The second part proposes a rationale for university responsibility to solve the problems of impoverished populations at a geographical remove. It also presents a framework for integrating science, engineering and ethics in the efforts of multidisciplinary teams dedicated to this task. The essay’s third part illustrates the efforts of Howard University researchers to join forces with African university colleagues in fleshing out a model for sustainable and ethical global development.

## Introduction

Throughout the literature of development, researchers focus on a variety of development ethics and dilemmas (Gasper and St. Clair 2010). They explore the definitions of community capability and democracy as instruments for achieving well-being (Sen 2000; Crocker 2009, 2012). They also examine conflicts between global and local ethical standards (Gasper 2004). They consider whether moral obligations to the needy decrease as a function of distance between the wealthy and the poor (Chatterjee 2004), and they address bad governance and other seemingly unethical underpinnings that explain why the poorest countries of the world, the “bottom billion,” seemingly do not advance economically despite global development initiatives (Collier 2007).

These examples of development ethics literature ground themselves in arguments on behalf of virtue ethics, deontology or utilitarianism. Resting on two assumptions, survival ethics is critically different as we define it. First, ethics arise as responses to crises. Second, the more threatening the crises, the more urgent the need for a collective, consensual response. Potential global threats to life through global climate change, enhanced weapons of mass destruction, and the misery of billions in the Global South prompt the need for a survival ethics model that grounds itself first and foremost on a single principle: to be good is first to be. It is not possible to be ethical unless one exists. Only after survival is assured can one afford to deliberate about abstract ethical and moral issues.

Our survival ethics model targets extremely poor populations, those who are chronically poor and live below the $1.25 per day poverty line (based on 2005 prices) or in some countries below the $2 a day median poverty line (USAID 2012; Chen and Ravallion 2012). It is the reality and voices of the poorest of the poor that should matter most in the developing world, yet it is their continued existence that poses the most difficult development challenges. Estimates of the world’s population that lives in dire need vary in the literature, generally being estimated at one to three billion people. How can we ensure the survival of these specific populations? Poorly-conceived development projects may cause more harm than good or fail to reach the most impoverished communities (Collier 2007).

The primary instrument of the survival ethics model is a checklist system that enumerates the basic conditions for survival—clean air, temperature control through clothing and shelter, potable water, nutritious food, basic healthcare and education. The optimum guarantee of survival is through pursuit of the conditions for flourishing—rationality through continuing education, heightened community bonding, intelligent use of pleasure as a mechanism for guiding behavior, freedom defined as the potential for creativity, and introspection defined as the rational control of attention (Verharen et al. 2011; Verharen 2008). Classical African, Asian and European philosophers have designated each one of these conditions as a *summum bonum* for humanity. Survival ethics regards all five as indispensable to flourishing. The relative importance of each condition is a matter for decision in the context of local cultures. Maximum cultural variation is a precondition for the survival of the species (Locke 1989).

Our model entails a global network of research universities working together with government agencies, non-government agencies, small and medium enterprises, community-based organizations, and most importantly the vulnerable populations themselves. Together, they can drive towards the ethical, sustainable development of communities at risk.

Survival ethics embodies an emerging network of international centers dedicated to sustainable development grounded in social justice. The objective of the network is to demonstrate that ethical development can achieve sustainable results. We demonstrate this through collaborative multi-disciplinary efforts focused on the implementation and evaluation of appropriate technology development projects. The model provides the flexibility to address challenges arising from the changing nature of the international development landscape. It also speaks to global trends in environment, culture, demographics, urbanization, economics, connectivity, healthcare, science and technology. One issue in development is the difficulty in isolating causal factors that affect the success of projects. We offer a program to determine what works and what does not work in this regard. The use of checklists is critical to the design, implementation and assessment of development projects.

Both survival ethics theory and the partnership model conform to goals sanctioned by United Nations (1948) Universal Declarations of Human Rights and the 2000 Millennium Development Goals. They also recognize the centrality of a community’s right to determine its own survival strategy and to maintain its own identity and culture—both tangible and intangible (Silversmith and Ruggles 2004; Singer and Stavenhagen 2004; Sen 2000). The Universal Declaration of Human Rights, Article 25, Section 1 (United Nations 1948) states the basic conditions for survival: “Everyone has the right to a standard of living adequate for the health and well-being of himself and of his family, including food, clothing, housing and medical care and necessary social services, and the right to security in the event of unemployment, sickness, disability, widowhood, old age or other lack of livelihood in circumstances beyond his control.” Article 26, Section 1 states the right to a basic education: “Everyone has the right to education. Education shall be free, at least in the elementary and fundamental stage”.

Article 26, Section 2 expresses the right to an education that promotes flourishing in the entire human community: “Education shall be directed to the full development of the human personality and to the strengthening of respect for human rights and fundamental freedoms. It shall promote understanding, tolerance and friendship among all nations, racial or religious groups, and shall further the activities of the United Nations for the maintenance of peace.” Principles fostering survival and flourishing define the basic philosophy of the UN Millennium Development Goals (United Nations 2000).

## Part I—University Responsibility for Ethical Development: Historical Context

In this part we briefly review the historical context in which universities have entered development initiatives and addressed ethical challenges. Academics have increasingly recognized the importance of their responsibility for alleviating and preventing global poverty (Bacon et al. 2013; Hutson 2013). Institutions of higher learning have been at the forefront of efforts to determine the directions of ethics-based development. Universities have played major roles in building better explanations of cultural behavior in order to pose more ethical and sustainable solutions for those in need (Stephens et al. 2008; Holmberg et al. 2013).

A historical perspective illustrates how academic institutions have embedded ethics into their applied work. By examining relationships between institutions of higher education, their applied research relating to international development and poverty, and the local communities affected, we can see how academics have increasingly seen a need for constructive evaluations of their collective impact on communities. We can observe how academics have grown to recognize the need to create clearly defined ethical codes integral to their responsibilities for the people that they are trying to help (Biedenweg et al. 2013; Byrne 2012; El-Zein et al. 2008).

Prior to the Great Depression, three main endeavors stimulated the growth of problem-oriented research: (1) interdisciplinary programs; (2) international research; and (3) an increased presence of the academy in social development. Academic institutions increasingly lobbied for enlarged responsibilities with respect to development and policy at the community, national, and international levels. The involvement of social scientists in evaluating the outcomes of development policies increased in the government, philanthropic institutions, and other organizations devoted to understanding global poverty, health, and conflict (Collier 2007).

Ethical concerns led to a focus on those working with local communities internationally, while at the same time compiling data for organizations whose agendas failed to protect the best interests of those same communities. On December 20, 1919, the founder of American anthropology, Franz Boas, accused some anthropologists of being spies and wrote in *The Nation*, “A person, who uses science as a cover for political spying … prostitutes science in an unpardonable way and forfeits the right to be classed as a scientist” (Price 2000). Boas’ comments set a mandate for academics to take the lead in establishing ethical approaches to working with affected communities.

In the 1930s and 1940s, historical events such as the Great Depression, the New Deal, and World War II shaped development work in institutions of higher learning and international development. During this time, many academics conducted research to address the harsh conditions of Americans as well as other vulnerable populations around the world. As more researchers worked in international settings, more situations highlighted the need to evaluate impacts on communities and address ethical concerns.

A graphic example may be cited from the anthropological work of Cora Du Bois. Funded by the Social Science Research Council, she conducted work with the people of Alor in what is now Indonesia. Prior to meeting Du Bois, the Alorese had never heard of the United States. As a result of her research, she developed rapport with them. During World War II when the Japanese occupied Indonesia, Japanese officers overheard several Alorese state that they wished Americans would win the war because they were good people. Du Bois later learned that they were publicly beheaded for their comments (Du Bois 1961).

Ethical discourse continued into the mid- and late 1900s, and during this period the arena of social development expanded. President John F. Kennedy established the US Agency for International Development (USAID) in 1961. The World Bank established the Safeguard Policy Sector specifically to address the impacts of development on indigenous peoples, their cultures and environment. Universities created programs devoted to area studies, modernization, and development theories. Concern over ethics in particular escalated as a result of major projects in the 1960s and 1970s.

One example is Project Camelot, funded in 1964 by the US Army’s Special Operations Research Office. This project was one of the largest grants for social science research then established. Its focus was on predicting the potential for conflict, and designing war prevention procedures for governments. In response, many scholars adamantly criticized the use of research to maintain political agendas that could lead to serious repercussions for oppressed classes and vulnerable populations. Reactions prompted widespread attention to ethics and resulted in the creation of committees devoted to the evaluation of unethical behavior (Horowitz 1967).

These events resulted in greater university attention to the harm that could come to vulnerable populations from unethical development. Historical lessons have established a springboard for the design of a development model based on survival ethics—the premise that regardless of political affiliation, socio-economic class, or national, religious or cultural identity, people all over the world have a basic right to survive and flourish.

## Part II—Integrating Science, Engineering and Ethics

By foregrounding the earth’s populations whose survival is at risk, we hope to encourage global university commitment to ethical and sustainable development issues. By addressing the misery of billions in the Global South, we endeavor to recruit students and engage young researchers who care deeply about the needs of the world’s deeply impoverished. What are the ethical foundations for this research enterprise?

Unlike deontological and utilitarian systems, survival ethics does not presume that ethics must be grounded in an assumed a priori universal law such as the categorical imperative or an a priori assumption that all humans must act for the greatest good for the greatest number. Ethicists like Joyce (2001, 2006), Hauser (2006) and Harris (2010) assume that humans care for one another as a result of natural selection. Wilson (2012) makes the provocative claim that humans by their biological nature are *eusocial*, or capable of sacrificing their personal interests for the sake of their groups in the very best of circumstances. Pinker (2011) makes the claim—counterintuitive to many—that rates of violence have declined markedly over the millennia because of advances in ethics.

A critical question for a survival ethics is why those whose survival is assured should concern themselves with others whose survival is at risk (Singer 2011). Like a feminist ethics of caring (Held 2005; Noddings 2005; Slote 2007), survival ethics reaches out to those who are moved to address these problems. The argument we urge on behalf of survival ethics is a pragmatic one. Our quality of life is by and large dependent on the quality of our groups. In the short run we may enhance our personal quality of life by acting against the interest of our groups. However, in the long run it is in the interest of individuals to act on behalf of their groups. A group’s well-being depends in part on three factors: its size, the strength of its binding principles, and the excellence of each of its members.

Virtually all competitive current ethics systems assume that the concept of “our group” should include every human being. The binding principles of those systems require self-sacrifice for the sake of the group—in the extreme case giving up one’s life for the sake of the group. Excellence of group members is a function of their access to the conditions for survival and flourishing. Survival ethics appeals both to an innate sense of caring and to a self-interested conviction that working toward the interest of the group enhances personal well-being.

Survival ethics promotes the synthesis of science, engineering and ethics. In support of Blackburn (2001) and Singer (2011), and against Harris (2010) and Joyce (2006), we affirm the gulf between science and ethics. Modifying Sellars’ definition of philosophy (‘philosophy studies how things in general hang together’), we claim that science studies how things are and ethics considers how things ought to be. As Blackburn puts it, the mind has two distinct tasks: to understand and to desire. The former makes science possible and the latter makes ethics possible.

Ethics sets guidelines for the direction of life. Renowned ethicists have singled out distinctive goals: Hindus’ *moksha*; Buddhists’ *nirvana*; Plato’s knowledge of perfect forms; Aristotle’s *eudaimonia*; Christ’s universal, unconditional love; Hume’s emotions; Kant’s good will directed by reason; Hegel and Marx’s freedom; Nietzsche’s will to power. Scientists and engineers develop systems for realizing these goals. The objectives of ethics are categorical: each ethicist proclaims the priority of her particular goal. The objectives of science and engineering are hypothetical: if you wish to pursue this particular goal, then here are the steps you must execute. The conditions for survival are most obviously the concern of science, engineering and technology: what must we do to secure clean air, adequate clothing and shelter, pure water, nutritious food and basic healthcare and education?

Science, engineering and ethics are interdependent and co-evolutionary. Science (including both the natural and social sciences) describes things, presents explanations for why things exist, and offers evidence for predictions of the future. Ethics prescribes how things should be. And *engineering* in its oldest etymological sense of “giving birth” describes how to get from the way things are to the way things should be. Taken together, the three fields constitute a new multidisciplinary field that might be called *teleonomics* or *teleologistics* inasmuch as its aim is to reach desirable ends or goals (from the Greek *telos*, end) through appropriate means. Biologists have appropriated the first term for studies about questions of purpose in nature. Growing consensus on the futility of such questions (*pace* Nagel 2012) might lead us to “re-purpose” the term.

We cannot know how to pursue science and engineering without understanding how they fit into our goals for life. And our goals for life change with advancing knowledge in science and engineering. Due to the complexity of human behavior, analysis of the ethicality of means/ends relationships requires interdisciplinary research. If we say that x is good because it will produce y, then the context of the claim dictates the kind of research needed to substantiate the claim. If we say that a development project will guarantee the survival and enhance the quality of life of a community, the complexity of the project will dictate the numbers and professional competencies of the team assigned to evaluate the project in terms of the survival ethics checklists (Verharen et al. 2011; Verharen and Tharakan 2010).

Even a simple act like digging a well calls for a broad range of expert knowledge. How deep must it be? What must the slope be to ensure that the sides do not collapse? What is the expectation for the quality of water from the well? What levels of arsenic or salt are found in the water table? What is the projected depth of the well? Does the community have the resources to cover the cost of the well? Will the well be an excessive burden on an already stressed water table? Will the well displace the workers who make their living by carrying water from more distant sites? What is the projected life of the well? What effect will the well have on the social fabric of the community? What will the impact of the well be on surrounding communities?

To cite a graphic example, Bunker Roy’s assistant Ramniwas tells the story of his first encounter with the Barefoot College organization in Rajasthan, India. A Barefoot College well-drilling team saw the greatest need for a well in Ramniwas’ village, an outcaste community situated near a higher status village. Those higher status residents fiercely protested the well’s being drilled in the poor village. Both parties saw the issue as one of justice. The higher status villagers believed that their caste privileged themselves over outcastes. The Barefoot College drillers believed that the well would increase the morale and economic viability of the poorest residents of the area. Their vision of justice triumphed, but a better resolution of the conflict might have come from a survival ethics team approach (Barefoot College 2012).

Survival ethics teams form themselves around a constellation of research questions. For the technical aspects of such a project, geologists, biologists, archaeologists, environmental anthropologists, chemists, engineers and technologists all have roles in decision and evaluation processes. For the social science aspects, economists, political scientists, psychologists and cultural anthropologists join the conversation. For the humanistic aspects, historians, philosophers, linguists, classicists and artists make their contributions.

In the interest of increased objectivity, NGOs (Non-Governmental Organizations) add their expertise. Pursuing sustainability for the project includes participation of SMEs (Small and Medium Enterprises) that advise community members on forming a corporation for the maintenance of the well and for replacement wells in the long run. Most important is the participation of community members, both as individuals and as members of CBOs (Community Based Organizations). Here students are called upon to exercise service-learning initiatives. In collaboration with community members in rural areas and university experts, all-inclusive survival ethics teams strive to achieve culturally sensitive and ethical solutions to development challenges.

The US Agency for International Development (USAID) recently issued a Request for Applications (RFA) for well-funded partnerships in a “Higher Education Solutions Network” (USAID website). With this RFA the agency recognizes that historical development models often produce results that are either unsustainable or less than optimal for the survival and flourishing of communities selected for development projects. In some cases, ill-conceived projects have exacerbated the challenges for communities, contributed to further impoverishment, or have led to the subsequent destruction of other resources within local environments.

Embracing new approaches to ongoing development challenges, the agency is soliciting partnerships with universities in recognition that their research capacities can produce innovative models for sustainable development. The RFA is a tacit acknowledgement of the fact that universities have moral obligations to their constituent communities. Where universities are favored with abundant resources for research and a student corps for service-learning endeavors, those obligations extend beyond the bounds of national territories to those communities whose survival is most at risk.

## Part III—A Global Network for Survival Ethics

Given our ability to employ the talents of a wide array of academics and partners, we present a working model for development based on the concept of survival ethics. Research on its compatibility with ethical systems of traditional cultures in the Global South is critical to the success of this model (Gutema and Verharen 2012; Verharen 2012). Howard University researchers and colleagues from African institutions are drawing upon mutual global expertise in addressing the long-standing failures of global development projects. The Howard model builds on a network of university-based centers for ethical and sustainable development with an initial focus on Africa. While our goal is to bring together many different partners both inside and outside of Africa, as Howard University is an HBCU (Historically Black College and University), we also strive to enhance the networks and harness the resources of the Black Diaspora and impoverished African communities).

Due to the high rates of poverty and development challenges throughout Africa, strategic economic development is critical for this region. According to USAID (2012), three out of every four Africans live on less than $2 a day. Approximately half of all children who die are African. Africa is the only continent where crop yields have remained stagnant over the last forty years. Grounded in the concept of survival ethics, the Howard model establishes relationships between United States and African universities to address the health, technological, environmental, cultural, political and economic problems that cause poverty and the subsequent problems derived from poverty (e.g., human rights violations, conflict, displacement and marginalization).

In conjunction with the International Network on Appropriate Technology, Howard University has established partnerships with a cross-section of African universities in developing nations to leverage expertise in areas congruent with the core objectives of international development agencies. Virtual networks of indigenous and international experts collaboratively address challenges identified by communities and defined through assessment of collected data in targeted areas. Taking into account poverty indicators that define the most vulnerable communities is critical to the survival ethics protocol. The uniqueness of this approach is the establishment of student outreach programs in tandem with ethical, community-inclusive partnerships that are data-driven, evidence-based, and tied to the use of appropriate technologies. The model empowers communities to address self-prioritized needs, improve quality of life and raise standards of living.

### Survival Ethics Model

Like other initiatives in Africa that use science and technology to reduce poverty, this model partners communities with academia, NGOs, SMEs and CBOs (Zeng 2008). The survival ethics model harnesses the enthusiasm of youth in the US and developing countries. It directs the passions and focus of these team members in a framework with expert guidance and mentorship from academics and practitioners. It also serves to bring people together to implement innovative and sustainable solutions to community-identified problems.

The approach addresses community-delineated issues within an ethical, social and culturally aware decision-making framework that involves all affected groups and promotes gender equity. Full participation of team members and assessment and evaluation at all phases is also part of the model.

The survival ethics model includes the following:

- A core of interdisciplinary faculty trained in social and ethical concept and component inclusion in science and engineering education (Tharakan et al. 2005; Tharakan 2006).
- Faculty who recognize the potential of their students to promote community capacity-building (Tharakan et al. 2008).
- Networking among NGOs, SMEs, CBOs, community member partners, target organizations and individuals throughout the African Diaspora, and governmental agencies engaged in specific development projects—a synergistic outcome of faculty exchange and mentoring (Fortunak and King 2010).
- Revisions and inclusion of service-learning courses in technology curricula. Curricular offerings focus on: (1) ethics and the philosophy of technology and engineering; (2) appropriate technology for developing communities with focus on water, food security, sanitation, and environment; (3) alternative and renewable energy solutions in developing communities; (4) pharmaceutical manufacturing; (5) cultural and historical context, facilitation, and working with community members; (6) environmental, archaeological, and heritage conservation; (7) data collection, evaluation, assessment, and monitoring (Tharakan 2006, 2011, 2012; Tharakan et al. 2005; Bugarin 2009); (8) and socially relevant computing (Kadoda 2012).
- A new generation of students trained in socially and ethically responsible engineering and social development. Students gain real world experience in development through mandatory curricular engineering service-learning projects (Tharakan 2012).
- All teams learn from and work in tandem with the communities they hope to help (Fortunak and King 2010).

As a pragmatic approach to solving problems for populations in need, survival ethics benefits university faculty and students as well as community members. The survival ethics model teaches both students and faculty to deal with real-world issues in a real-world context (Balazs and Morello-Frosch 2013; Nussbaum 2013).

### Deploying the Model

**Fig. 1:**
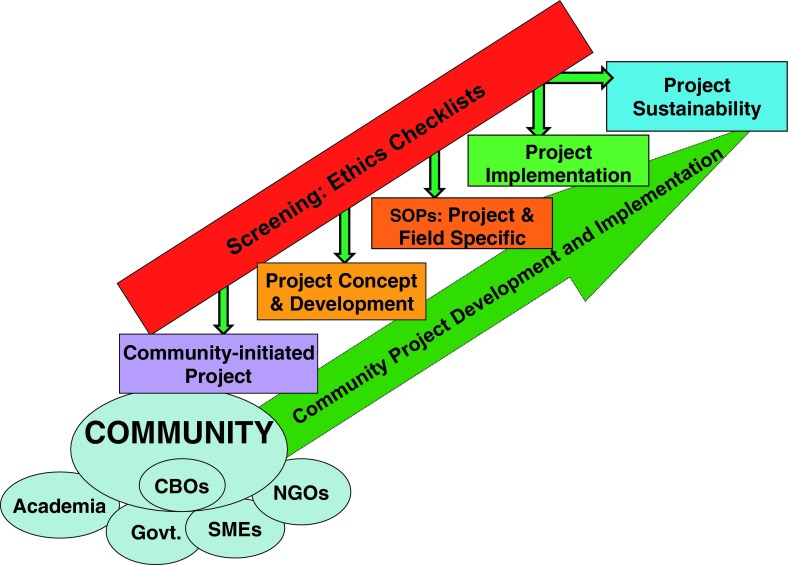
Model for generating an ethical, community-based developmental project through integration of a screening checklist approach in all phases of the project

In the initial efforts to execute the survival ethics model, community-based projects are designed, developed and implemented in partnership with NGOs, SMEs, CBOs and the inclusion of communities at all phases (Fig. 1). The potential for sustainability of these projects will be significantly higher, since monitoring and evaluation will be conducted on a long-term, on-going basis with community participation.

#### Community Health

We note the following example in East Africa. At the request of the St. Luke Foundation in Moshi, Tanzania, Howard and Purdue University researchers and African partners have established an Industrial Pharmacy Training Unit at the Kilimanjaro School of Pharmacy/St. Luke Foundation. The Unit teaches the fundamentals of drug development, regulation, and quality-assured drug production. World-class experts in drug discovery and development produced a curriculum that teaches attendees how to manufacture medicines efficiently and with assured quality. Contributors from the pharmaceutical industry, drug regulation agencies (USFDA), law firms (patents) and academics have further tailored the program for the needs of African pharmaceutical professionals. This program utilizes intensive classroom training, team exercises and hands-on product development in a laboratory and a pilot production facility that has been designed and built for this purpose.

Completing the program, participants are able to develop new drug products and processes, as well as meaningful tests and specifications to assure drug quality. Participants are also able to utilize their learned skills to detect substandard and counterfeit medicines. Participants also understand how to meet International Standards for Quality Assurance and are, therefore, prepared to make applications to sell their products to International Donor Agencies as one path for economic development. These operations are governed by a Quality Management System that includes roughly 400 Standard Operating Procedures to assure proper oversight of desired ethnical principles and congruence with international standards of quality assurance practice (Fortunak and King 2010).

The contribution of survival ethics to expanding global access to medicines is a useful, specific example. The lack of access to medicines greatly increases mortality and saps economic growth through associated morbidity in low- and middle-income countries (LMICs). Current life expectancy in the United States is approximately 78.1 years (US Life Tables 2008). In Nigeria, by comparison, current life expectancy at birth (2012) is about 52.05 years (Index Mundi).

Much of the difference in these figures can be attributed to the lack of access to modern medicines. Although high-income countries and medicines donation programs largely focus on the “big three” diseases of HIV/AIDS, malaria, and tuberculosis, many more people in Nigeria and other African countries die from simple infections, cardiovascular disease (i.e., hypertension, heart failure), diabetes and cancer than from these “big three.” The point to be made is not that high-income countries are “wrong” in donating medicines for HIV/AIDS, TB and malaria to LMICs, but rather that donation programs are not a comprehensive or sustainable solution to the overall problem.

Medicines are intended for human or animal consumption to treat disease. As such, a high level of ethical reflection goes into their production and management. Since 2000, substantial funding has been made available from governments, NGOs and Foundations to provide medicines to LMICs. The Global Fund for AIDS, Tuberculosis, and Malaria (GFATM), the US President’s Emergency Plan for AIDS Relief (PEPFAR) and the Bill and Melinda Gates Foundation each provide over $1 billion annually to promote access to medicines for AIDS, tuberculosis, and malaria. Substantial, overlapping contributions from science, engineering, and ethics are needed to enable sustainable access to affordable, high-quality medicines. The correct balance of application from all these areas assures the effectiveness, efficiency, and maximum sustainability of access to quality medicines for LMICs.

Survival ethics for medicines is a multi-faceted issue. Simply donating medicines to LMICs saves lives. Donations also, however, perpetuate a long-term culture of dependence and de-emphasize national sovereignty in drug manufacturing and regulation. A comprehensive approach to survival ethics involves developing the regional capacity to discover, manufacture, and regulate medicines. This (a) helps end the culture of dependence upon donations; (b) contributes to industrial and economic development; (c) increases access to medicines beyond those for AIDS, TB, and malaria; and (d) helps address the widespread problem of counterfeit medicines that largely result from unscrupulous importation and ineffective national regulation.

Our role in survival ethics for “Access to Medicines” includes enabling the regional manufacture of high-quality, critical medicines in a most affordable and environmentally benign manner. Our work also includes training national drug regulatory agencies in detecting counterfeit and substandard medicines. The ethical regulation and manufacturing of medicines is substantially dependent upon the development of human resources. National drug regulatory agencies and manufacturing companies must, on the whole, understand the elements of the following: (a) science; (b) Good Manufacturing Practice (GMP); and (c) quality management. The end result is that companies will consistently manufacture high-quality products, and drug regulators will protect their populations by identifying counterfeit and substandard medicines and assuring that companies selling medicines are regularly capable of meeting required standards.

With this approach, we have trained over 80 pharmaceutical and regulatory professionals who have contributed to the approval (by the US FDA or the WHO Pre-Qualification of Medicines Program) of three African companies to sell their medicines to donor agencies, thereby promoting economic development and increased access to medicines in Sub-Saharan Africa.

#### Clean Water Supply

Engineers Without Borders-USA (EWB-USA) is a US-based international service organization focused on projects in developing communities that address basic needs congruent with the survival ethics model. EWB-USA has numerous professional and academic student chapters across the country and the world. The student chapters, under the guidance of academic advisors and professional mentors, partner with communities in developing countries and work with them to address community-prioritized needs.

The Howard University Student Chapter of EWB (EWB-HU) has engaged in a long term partnership with the Choimim community and the Build the Village NGO in the Nandi Hills region in northwestern Kenya, working together with the community to develop and enhance pressing quality of life issues around the critical resource of water. The community had identified water quantity and quality as its two most pressing concerns. Responding to these needs, the EWB-HU chapter implemented rainwater harvesting (RWH) to increase water storage capacity in the community, and installed freestanding bio-sand filters (BSFs) to increase community capacity for potable water production.

In the assessment and implementation process, students worked under professional and academic guidance in a service learning context. Community recipients of the BSFs were trained as community engineers (CEs) and tasked with operation and maintenance of BSFs. In addition, the student chapter was able to engage a local NGO to ensure local sourcing of all materials and to develop a monitoring and evaluation (M&E) plan that includes regular testing of the BSF for flow (capacity) and water quality. Working with the NGO, the CEs also make the BSF technology available to surrounding communities (Water Is Life Project Website).

#### Gender Equity

We note the following example of the model’s deployment in Sudan. Under the auspices of the International Network on Appropriate Technology, Howard University and University of Khartoum faculty promoted a partnership with the Barefoot College in Rajasthan, India. The College’s training activities equip the rural poor with technical skills to provide themselves with in-ground water storage tanks and solar electrification. The College trains women from remote villages in Asia, Africa and Latin America over a 6 month period. The Barefoot College’s partnership model brings together a local NGO and a local community.

The NGO locates a village, monitors project progress, and ensures that the selected community is engaged during candidate selection, project planning and implementation. Training preference is given to non-literate or semi-literate older women (young grandmothers) who are unlikely to leave their villages. The agreement established at the project onset states the financial contribution of the village towards the wages of the returning trainees and for future material needs, while the College secures start-up funds through agreements with organizations that operate small-grant programs in the country.

Two villages in the Nuba Mountains in Sudan were selected because they were far from the electrical grid and because their members were enthusiastic about the projects. The phases of the project included constructing a village workshop (solar engineers working space) from local material, solar electrifying two villages (Mirri and Aldorot), and establishing a local awareness and training program with the vision of spreading the project to nearby villages and to other parts of the country.

The recent war in the Nuba Mountains destroyed the villages. Two of the solar engineers returned to the Barefoot College in Rajasthan to join the September 2012 solar electrification course to retain their currency. Their training keeps them up to date for rebuilding when the time is right, and enables them to act as Trainers-of-Trainers, replicating the Barefoot College experience on a local scale. By the time Mirri village was solar electrified, there were nine community members who had become apprentices to the solar engineers.

Various elements of the Barefoot model are related to the survival ethics approach. Most prominent are female empowerment, appropriate technology for the environment, the partnership of community members, as well as NGO, CBO and academic activism in the development process. One of the solar engineers applied a local ethical principle of “the sacredness of something one is entrusted with” to the knowledge she acquired from training to deliver to her community. This sacredness sustains the project through the College’s selection criteria of what is most vulnerable—community—and least acknowledged—women. The College defines success through a community perspective rather than by traditional certification.

Many in the development world have recognized the need to address gender issues as they pertain to international development, poverty, ethics and science (e.g., Malhotra and Schuler 2005; Campion and Strum 2004). Building upon their contributions, the survival ethics model stresses the importance of gender equality and female empowerment. Addressing the needs of both genders, our strategy entails: (1) advocacy and female mentorship pairings; (2) participatory appraisals and social impact assessments that engage both genders; (3) facilitating leadership roles for both women and men; and (4) development of gender appropriate training and educational tools. Underlying each initiative is an ethical core that promotes trust between genders to build rapport.

The foundation of ethical development relies on confidentiality, consent, and risk disclosure. To do this, we obtain consent from participants and facilitate environments that guard their confidentiality. We include both genders in the decision-making process. In addition to all-inclusive community meetings, we meet separately with women and men to encourage further input. Assessment, performance evaluations, and documentation of communication challenges will gauge our abilities to engage both genders.

#### Natural and Cultural Environment

We include the following example to illustrate the independent application of survival ethics principles to problems of ethical, sustainable development in Latin America. In 2008 the government of Ecuador implemented an innovative national forest conservation program to benefit landowning indigenous communities—the *Programa Socio Bosque*. Funded by public fees and Reducing Emissions from Deforestation and Forest Degradation (REDD) carbon emission reduction funds, the program conserves over 5 million hectares of forested land and provides income to 2 million mostly poor indigenous people and farmers.

The program also serves to reduce illegal logging and agricultural-induced deforestation. The program recognizes the role of these communities in conservation, providing direct income in exchange for commitments to protect key areas of forest in places where at least 50 % of the population is below the poverty line and where important ecosystems are not currently included in the national protected area system.

Communities are involved at all levels of decision-making, including decisions whether to participate or not. Besides implementing a domestic incentive-based policy to tackle deforestation, the program offers a clear and transparent mechanism for delivering benefits to local communities, including indigenous peoples (de Koning et al. 2011).

As development literature suggests, the protection of biodiversity in tandem with the conservation of the built cultural environment and social development is crucial (MacKinnon et al. 2004; Pierce et al. 2002; Bugarin 2009). To ensure both food and social security, a comprehensive environmental and landscape analysis must attend to natural and cultural resources as well as farming system, agronomics, soil analysis, ecological systems and natural resource management (e.g., foraging and pastoral strategies). Our dedication to environmental sustainability within a cultural landscape is woven into all activities through: (1) studies of subsistence strategies focused on achieving food security; (2) assessment, mitigation, and monitoring of all project phases; (3) coordination of experts; (4) explicit protocols; (5) community-based initiatives; and (6) teaching, training, research and outreach.

Projects are developed with an all-inclusive approach to ensure that both natural and cultural environmental resources are safeguarded, particularly while alternative subsistence strategies are devised for communities lacking food security. Following those who recognize that sustainable social development mandates attention to heritage sites, cultural and natural resources, and the ways in which humans interact with a variety of resources in their landscape to meet their basic needs (Breen 2007; Samuels 2007), we recognize that sustainability must take into account a wide array of variables. In addition, we strive to ensure that environmental hazards are not generated, and if they are, that they are handled in accordance with appropriate regulations, guidelines, and criteria.

Environmental sustainability will be ensured through data-driven initiatives, conservation efforts, and preservation. Archaeological, soil, water, botanical and faunal data are collected from communities to build an environmental database, identify resources, define patterns of diet and ecological strategies and pinpoint harmful activity areas. Information regarding biodiversity, the habitats of endangered species, and various ecosystems will also inform projects. When applicable, biologists and environmental anthropologists will assess development impacts on communities of both wild and domesticated animals, cultivated lands, soils, natural water resources and native plant species.

Through archaeological and heritage conservation efforts, we also identify disposal patterns, ecological relationships, and impacts through surveys, interviews, excavations, maps, and ethnographies. Aerial photography, archaeological data, and the use of Geographical Information Systems (GIS) allow us to analyze long-term impacts on a landscape, chart environmental changes, pinpoint untouched fertile areas for potential cultivation and create visual tools and maps to devise resource management strategies. These strategies are particularly useful where they inform conservation projects, land management plans and socially relevant computing approaches in engineering and technology.

#### Model Assessment

Howard’s partnerships with institutions in Ethiopia, The Gambia, Ghana, Kenya, Senegal, South Africa, and Tanzania serve as foci for testing the survival ethics model. Each partnership focuses on incorporation of social and ethical engineering education and practices to make critical and sustainable impacts on development challenges. They employ the model of community partnerships with multidisciplinary students and faculty, along with NGOs and SMEs to create and implement development solutions. Our expectation is that the successful execution of the model will provide development agencies with a clear ethical framework for the determination of aid needs and priorities. Anticipating success, we will endeavor to extend the model to the rest of the world, particularly the Caribbean, Latin America and impoverished regions of Asia and Oceania.

Critical to the success of the survival ethics protocol are effective measures of assessment and evaluation, as crafted by many in the development world. The survival ethics model focuses on five areas of assessment: (1) the ethics-based checklist (Verharen et al. 2011; Verharen and Tharakan 2010); (2) participatory rural assessments and community-based social assessments (Tharakan 2012, 2011); (3) the integration of faculty and students in service-learning, social development, and engineering programs with NGOs, SMEs, and CBOs (Fortunak and King 2010); (4) natural and cultural environmental impacts, including impacts on agriculture and other types of subsistence strategies (Bugarin 2009, 2002); and (5) gender and child impacts (Malhotra and Schuler 2005).

Partnerships in development projects are assessed by the rate and efficiency of their establishment, their relationships with NGOs, SMEs, CBOs, and their ability to meet the needs of the poorest communities. Evaluation of community-based projects examines the inclusion of the community in all phases of the project from conceptualization and prioritization of needs to project design and implementation. The potential for sustainability of these projects is determined through effective monitoring. In addition, the effectiveness of knowledge and technology transfer is examined with respect to informed community engagement, discussion and approval, quality of community outreach, education, capacity building, extension beyond the community, and evidence of government and multilateral aid agency support.

## Conclusion

Survival ethics teams have the expertise to pinpoint causes of inefficient subsistence strategies, document environmental features, plot landscape transformations, identify pollutants, predict impacts, and devise effective mitigation policies. We have the equipment to conduct archaeological excavations, soil and bone chemistry analysis, farming systems analysis, water quality assessments, tests for food-borne illnesses caused by natural resources, and investigations of air quality. Environmental education and outreach are an integral component of all projects. We train students and communities, consult with NGOs and SMEs, and raise awareness of environmental control technologies. Standard Operating Procedures are used to manage key personnel who handle waste products or engage in practices that impact the environment. Partners and communities are educated about potential dangers and contaminants and made aware of the benefits of a clean and safe environment.

An ethical model of sustainable development is grounded in a multi-disciplinary framework. It leverages the expertise of academics, SMEs, and NGOs, while it relies on the energies and talents of young students. Most importantly, it is community-driven and recognizes the importance of empowering communities that are able to maintain their own solutions.

Two open questions remain in this philosophical approach to ethical, sustainable development. First, will long-range assessment of ethics-based development strategies demonstrate their superiority to other models? Second, is it possible to scale ethics-based development to global applications? Gawande (2009) along with others is a proponent of a “checklist model” to ensure quality care, decreased costs, and constant innovation of hospital care. He proposes scaling up the checklist model to whole surgical teams including pre- and post-operative personnel, as well as to hospital consortia (Gawande 2012). Successful experimental results with comparable models (Fortunak and King 2010) encourage the authors to pursue broader application of the survival ethics checklist model (Verharen et al. 2011; Verharen and Tharakan 2010).
